# Quantification of peri-aortic root fat from non-contrast ECG-gated cardiac computed tomography

**DOI:** 10.1016/j.dib.2015.11.022

**Published:** 2015-11-19

**Authors:** Chun-Ho Yun, Chris T. Longenecker, Hui-Ru Chang, Greta S.P. Mok, Jing-Yi Sun, Chuan-Chuan Liu, Jen-Yuan Kuo, Chung-Lieh Hung, Tung-Hsin Wu, Hung-I Yeh, Fei-Shih Yang, Jason Jeun-Shenn Lee, Charles Jia-Yin Hou, Ricardo C. Cury, Hiram G. Bezerra

**Affiliations:** a Department of Biomedical Imaging and Radiological Sciences, National Yang Ming University, Taipei, Taiwan; b Department of Radiology, Mackay Memorial Hospital, Taipei, Taiwan; c Division of Cardiology, Department of Internal Medicine, University Hospitals Harrington Heart & Vascular Institute, Case Western Reserve University, Cleveland, OH, USA; d Institute of Health Policy and Management of Public Health, National Taiwan University, Taipei, Taiwan; e Department of Public Health, College of Public Health, National Taiwan University, Taipei, Taiwan; f Department of Electrical and Electronics Engineering, Faculty of Science and Technology, University of Macau, Macau, China; g Graduate Institute of Health Care Organization Administration, College of Public Health National Taiwan University, Taipei, Taiwan; h Health Evaluation Center, Mackay Memorial Hospital, Taipei, Taiwan; i Department of Medical Technology, Yuanpei University of Science and Technology, Hsin-Chu, Taiwan; j Division of Cardiology, Department of Internal Medicine, Mackay Memorial Hospital, Taipei, Taiwan; k Department of Medicine, Mackay Medical College, and Mackay Medicine Nursing and Management College, New Taipei, Taiwan; l Cardiovascular MRI and CT Program, Baptist Cardiac Vascular Institute, Miami, USA

## Abstract

In this data, we present the details of the cross-sectional study from Mackay Memorial Hospital, Taipei, Taiwan that examined the relationship between three-dimensional (3D) peri-aortic root fat (PARF) volumes, cardiometabolic risk profiles, carotid artery morphology and remodeling. Our sample is composed of a total 1492 adults who underwent an annual cardiovascular risk survey in Taiwan.

PARF was measured using images of gated non-contrast cardiac computed tomography (CT) and a dedicated workstation (Aquarius 3D Workstation, TeraRecon, San Mateo, CA, USA). The stratified analyses were performed in order to assess the association between carotid morphology, remodeling and PARF by tertile. For further analyses and discussion, please see “The Association among Peri-Aortic Root Adipose Tissue, Metabolic derangements and Burden of Atherosclerosis in Asymptomatic Population” by Yun et al. (2015) [1].

**Specifications Table**

**Table:** 

Subject area	Radiology
More specific subject area	Multi-detector computer tomography
Type of data	Peri-aortic root fat volume
How data was acquired	Multi-detector computer tomography (MDCT) (Sensation 16, Siemens Medical Solutions, Forchheim, Germany) and post processing workstation (Aquarius 3D Workstation, TeraRecon, San Mateo, CA, USA)
Data format	3D volume reconstructions
Experimental factors	MDCT imaging was performed as described [1]
Experimental features	Peri-aortic root fat was quantified using a post processing workstation (Aquarius 3D Workstation, TeraRecon, San Mateo, CA, USA)
Data source location	Taiwan
Data accessibility	Data are provided with this article

**Value of the data**

• Identifying the PARF measures associated carotid remodeling and morphology
• Provide the novel information about the difference between PARF and pericardial fat.
• These data can be useful in studies of cardiovascular risk assessment in similar populations, particularly for stroke and carotid artery disease.
• The data can be used to compare PARF, pericardial fat and cardiovascular risk in other populations.

## 1 Data, experimental design, materials and methods

### 1.1 Data

In this present data, we provide the details of the cross-sectional study that assessed the relationship between three-dimensional (3D) peri-aortic root fat (PARF) volumes, cardiometabolic risk profiles, carotid artery morphology and remodeling in a large group of adults. We present the baseline characterics of the samples, the assessment of carotid ultrasound and the association analyses between carotid intima-media thickness, remodeling, plaques and PARF by tertiles.

### 1.2 Experimental design, materials and methods

**Fig. 1:**
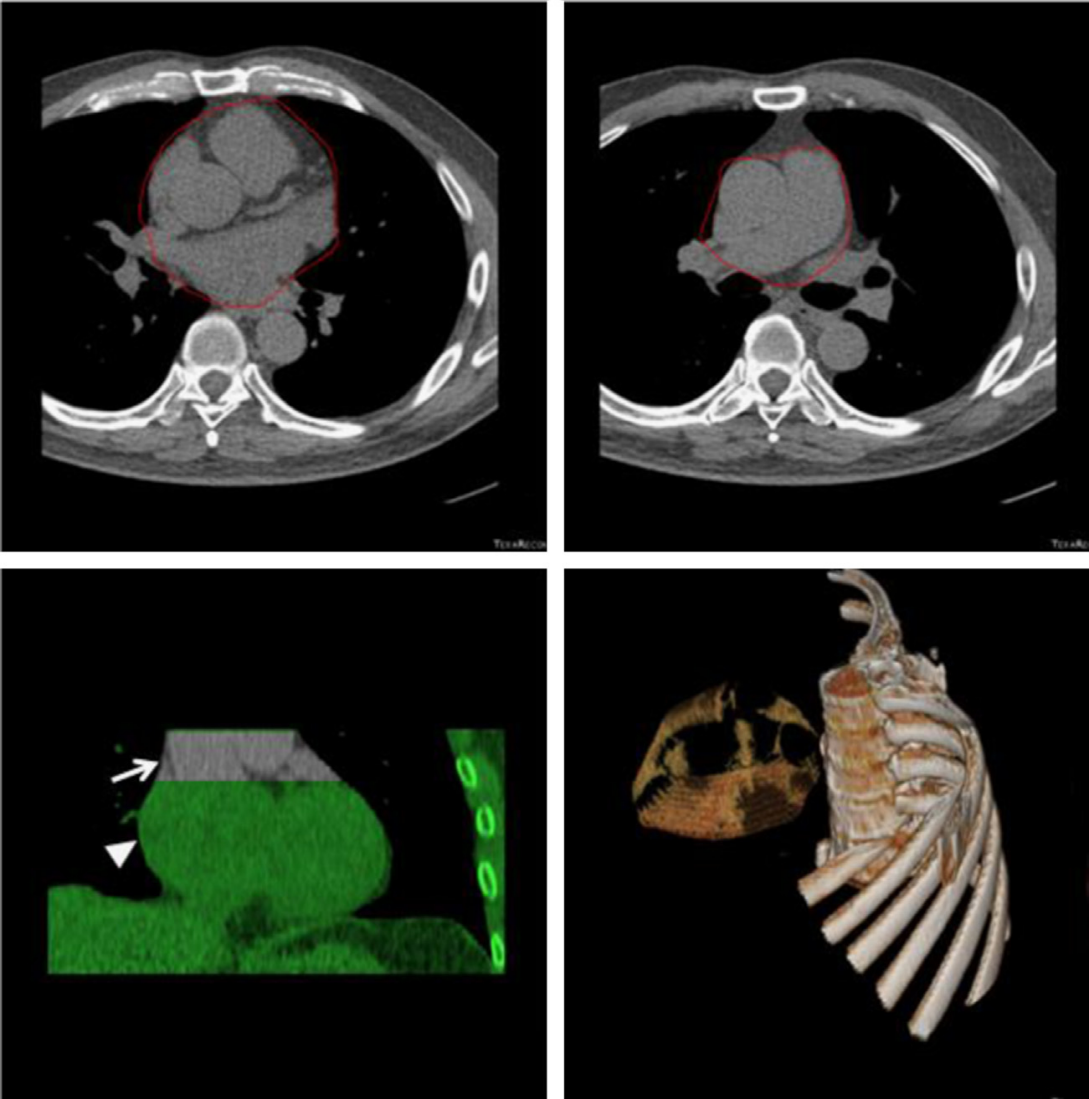
(a) Tracing of the pericardium (in red) at the level of left main coronary artery (LM) in the axial view. This is the caudal limit of peri-aortic root fat (PARF); (b) the pericardium is then traced (in red) in each of eight slices extending 24 mm (8×3 mm^2^ slices) cranially from the level of LM; (c) the cranial and caudal limits of PARF (arrow) are shown in the coronal view on the top of the pericardial fat and heart (arrow head); (d) three dimensional (3D) reconstruction of PARF, shown in relationship to the thoracic spine and ribs. The volume quantification of PARF of this case is 18.6 ml. (For interpretation of the reference to color in this figure legend, the reader is reffered to the web version of this article.)

**Fig. 2:**
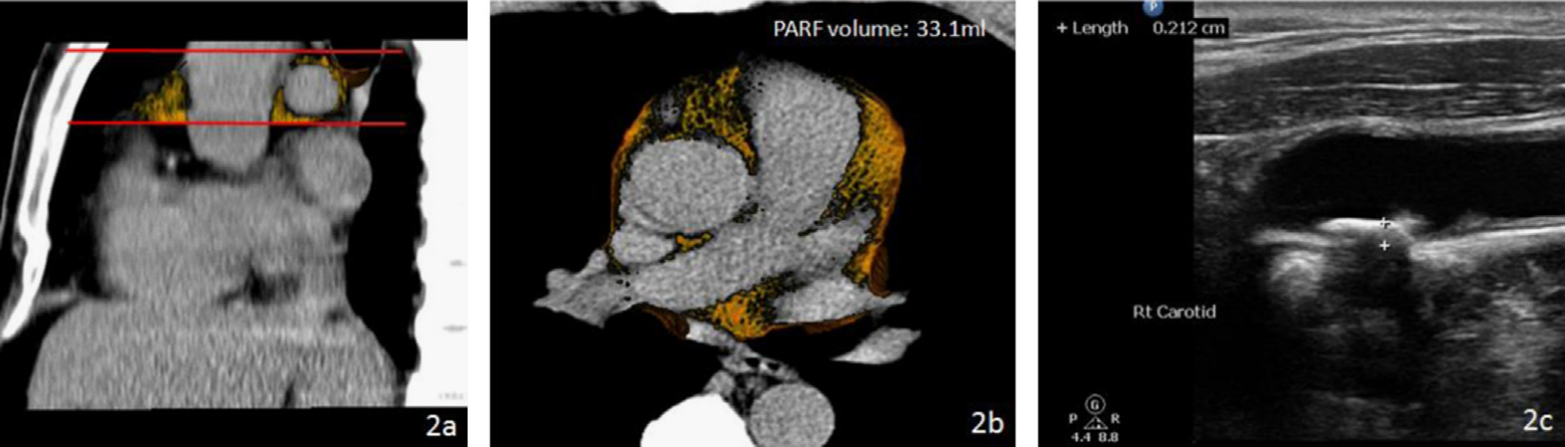
Comparison of two study subjects—one with a larger volume of peri-aortic root fat (PARF). Red lines indicate the 24 mm caudal to cranial limits of PARF. The orange color indicates adipose tissue surrounding the aortic root in sagittal (a) and axial (b) views. High resolution carotid ultrasound in the study subject with a larger volume of PARF (33.1 ml) showed greater intima-media thickness (2.1 mm) and plaque existence (c). (For interpretation of the reference to color in this figure legend, the reader is reffered to the web version of this article.)

**Fig. 3:**
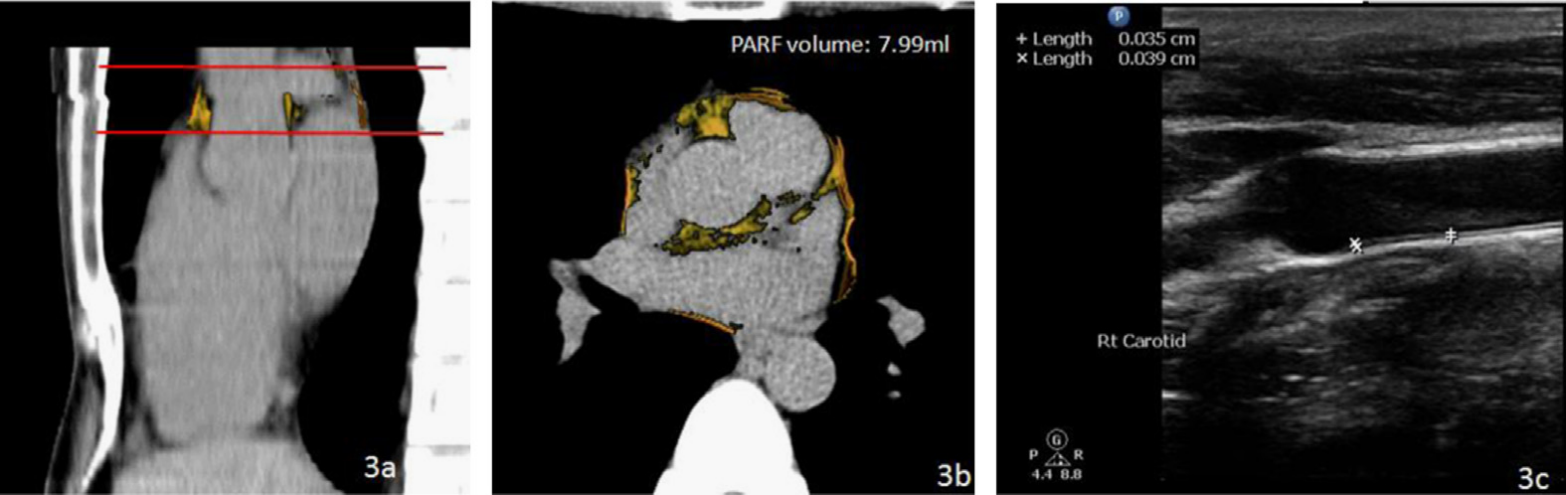
Comparison of two study subjects—one with a relatively small amount of PARF. Red lines indicate the 24 mm caudal to cranial limits of PARF. The orange color indicates adipose tissue surrounding the aortic root in sagittal (a) and axial (b) views. The relatively healthy subject with smaller PARF (7.99 ml) had thinner IMT and no carotid plaque (c). (For interpretation of the reference to color in this figure legend, the reader is reffered to the web version of this article.)

We have established a novel method to quantify the volume of peri-aortic root fat [1]. A total 1492 consecutive subjects who underwent annual cardiovascular risk survey in Taiwan were studied. Multi-detector CT (MDCT) of the heart was performed using a 16-slice scanner (Sensation 16, Siemens Medical Solutions, Forchheim, Germany) with 16×0.75 mm^2^ collimation, rotation time 420 ms and tube voltage of 120 kV. In one breath-hold, images were acquired from above the level of tracheal bifurcation to below the base of the heart using prospective ECG triggering with the center of the acquisition at 70% of the R–R interval. From the raw data, the images were reconstructed with standard kernel in 3 mm thick axial, non-overlapping slices and 25 cm field of view. All image analyses were performed on a dedicated workstation (Aquarius 3D Workstation, TeraRecon, San Mateo, CA, USA). Fat tissue was defined as pixels within a window of −195 to −45 Hounsfield Units (HU) and a window center of −120 HU. PARF borders were manually defined by tracing the pericardium on eight axial slices which extended cranially 24 mm from the level of the left main coronary artery. The predefined image display setting of fat was used to discern fat from the remain portions of other tissues of the aortic root level inside the pericardial sac, producing a cap of fat which is not only surrounding the aortic root but also on the top of traditional defined pericardial fat in our previous and Framingham studies [2], [3]. The volume of PARF was acquired from the sum of all voxels of fat and a subsequent 3D reconstruction was performed ( Fig. 1, Fig. 2, Fig. 3)
